# Research on the Correlation of Serum PCT and Plasma GSN Levels with the Prognosis of Urosepsis Patients

**DOI:** 10.12669/pjms.36.5.2143

**Published:** 2020

**Authors:** Na Cui, Zhanbiao Yu, Zhi Chen, Ning Chen

**Affiliations:** 1 Na Cui, Department of ICU, Affiliated Hospital of Hebei University, Baoding, Hebei, 071000, P. R. China; 2 Zhanbiao Yu, Department of ICU, Affiliated Hospital of Hebei University, Baoding, Hebei, 071000, P. R. China; 3 Zhi Chen, Department of ICU, Affiliated Hospital of Hebei University, Baoding, Hebei, 071000, P. R. China; 4 Ning Chen, Department of ICU, Affiliated Hospital of Hebei University, Baoding, Hebei, 071000, P. R. China

**Keywords:** PCT, GSN, Urosepsis, Prognosis

## Abstract

**Objective:**

To explore the correlation of procalcitonin (PCT) and gelsolin (GSN) with the prognosis of urosepsis patients.

**Method:**

The data of 71 urosepsis patients from March 2015 to April 2019 who were admitted to and treated in Affiliated Hospital of Hebei University were analyzed and compared with those of 92 healthy persons. Serum PCT and plasma GSN levels at different times after treatment were detected. According to prognosis, patients were classified into the good prognosis group or the poor prognosis group. The serum PCT and plasma GSN levels of both groups were compared.

**Result:**

The serum PCT level of the urosepsis group on the 1^st^, 3^rd^, 5^th^ and 7^th^ days was obviously higher than that of the control group (P<0.05). The plasma GSN levels of the urosepsis group on the 1^st^, 3^rd^, 5^th^ and 7^th^ days were obviously lower than those of the control group (P<0.05). The serum PCT level of the poor prognosis group on the 1^st^, 3^rd^, 5^th^ and 7^th^ days was obviously higher than that of the good prognosis group (P<0.05). The plasma GSN level of the poor prognosis group on the 1^st^, 3^rd^, 5^th^ and 7^th^ days was obviously lower than that of the good prognosis group (P<0.05). PCT was an independent risk factor influencing the prognosis of urosepsis patients and that GSN was a protective factor (P<0.05).

**Conclusion:**

The serum PCT and plasma GSN levels can accurately predict the severity and prognosis of urosepsis patients and reflect the disease state of early urosepsis patients. High PCT levels and low GSN levels indicate poor prognosis, and clinicians should consider these values.

## INTRODUCTION

Urosepsis is caused by urinary tract infections, with fast progress and a high fatality rate. Early detection and timely treatment are key in successfully controlling infection and lowering the death rate. The research shows that PCT can be detected in patients’ peripheral blood and hepatic tissues and can be used as a systemic infection diagnosis index.^1^ GSN is an important actin binding protein and plays an important role in cytoskeleton structural rearrangement, cell movement and programmed cell death control. The plasma GSN level of critically ill patients, such as those with serious septic shock, is substantially lowered and is closely related to patient prognosis.^2^ In this study, the serum PCT and plasma GSN levels of 71 urosepsis patients were dynamically observed to analyze the relationship between the two and the prognosis of urosepsis patients. In addition, prognostic risk factors of urosepsis patients were investigated.

## METHODS

The data of 71 urosepsis patients who were admitted to and treated in Affiliated Hospital of Hebei University from March 2015 to April 2019 were collected. Of the 71 patients, 15 had urinary obstruction with ureteral calculi. There were 43 male patients and 28 female patients. Their age was 18~65 years, with an average age of 39.11±7.32 years. A total of 92 healthy people examined within the same time period were included in the control group, including 51 male persons and 41 female persons. Their age was 18~65 years, with an average age of 37.62±6.95 years. Comparison of the general data of both groups, such as age and sex, revealed no statistical significance (P>0.05). This study was approved by the Medical Ethics Committee of our hospital, and all subjects signed informed consent. The inclusion criteria were as follows: (1) confirmed diagnosis of urosepsis according to the diagnostic criteria of the European Association of Urology (EAU).^3^; (2) age greater than 18 years but less than 65 years; (3) patient willingness to participate and to sign the informed consent form. The exclusion criteria were as follows: (1) presence of septicopyemia caused by other pathologies; (2) presence of malignant tumors, serious heart, liver, lung or kidney failures, or diseases of the immune system; (3) discontinuation of treatment before the study completion; (4) length of hospital stay<24 hour or survival time<72 hour; (5) history of immunosuppressive therapy within the previous three months.

### Ethical Approval

The study was approved by the Institutional Ethics Committee of Affiliated Hospital of Hebei University (No.: 2015021; date:2015-3-10), and written informed consent was obtained from all participants.

### Ethical Approval

The study was approved by the Institutional Ethics Committee of Affiliated Hospital of Hebei University (No.: 2015021; date:2015-3-10), and written informed consent was obtained from all participants.

### Sample collection and index evaluation

Venous blood (3 ml) was collected from urosepsis patients on the 1^st^, 3^rd^, 5^th^ and 7^th^ day of treatment and from the control group on the day of examination. Next, the blood was held at room temperature for 30 min and then centrifuged for 15 minutes at 3,000 r/min. The supernatant was taken and stored in a -80 °C refrigerator for detection. An enzyme-linked immunosorbent assay was adopted to determine serum PCT and plasma GSN levels. The serum PCT and plasma GSN levels of the urosepsis group were recorded on the 1^st^, 3^rd^, 5^th^ and 7^th^ day of treatment. According to their SOFA scores, patients were classified into the good prognosis group or the poor prognosis group. The serum PCT and plasma GSN levels of both groups were compared, and multifactor logistic regression analysis was conducted to determine different prognostic factors.

### Data Analysis

SPSS 21.0 statistical software was employed for data analysis. The data with a continuous normal distribution were expressed as the mean ± standard deviation (x ± s). An independent Student’s t test was used for intergroup comparisons, or data were expressed as the M±Q and tested with a nonparametric test. The enumerated data were expressed as a number (n; %) and tested via the χ^2^ test. A logistic regression equation was used for multiple-factor analysis. P<0.05 indicated that the difference was statistically significant.

## RESULTS

The serum PCT level of the urosepsis group on the 1^st^, 3^rd^, 5^th^ and 7^th^ days was obviously higher than that of the control group (P<0.05). The plasma GSN levels of the urosepsis group on the 1^st^, 3^rd^, 5^th^ and 7^th^ days were obviously lower than those of the control group (P<0.05), as shown in Table-I.

**Table-I:** Comparison of the serum PCT and GSN levels of both groups at different times (mg/L).

Group	1 day		3 days		5 days		7 days		n

	PCT	GSN	PCT	GSN	PCT	GSN	PCT	GSN	
Urosepsis group	6.17±1.03	182.13±19.18	6.07±1.14	142.37±21.19	5.37±1.15	101.27±17.36	2.93±0.97	73.48±11.35	71
Control group	0.29±0.14	289.41±39.72	0.29±0.14	289.41±39.72	0.29±0.14	289.41±39.72	0.29±0.14	289.41±39.72	92
t	26.07	25.83	26.99	31.43	24.72	39.25	22.38	47.97	
p	<0.001	<0.001	<0.001	<0.001	<0.001	<0.001	<0.001	<0.001	

The serum PCT level of the poor prognosis group on the 1^st^, 3^rd^, 5^th^ and 7^th^ days was obviously higher than that of the good prognosis group (P<0.05). The plasma GSN level of the poor prognosis group on the 1^st^, 3^rd^, 5^th^ and 7^th^ days was obviously lower than that of the good prognosis group (P<0.05), as shown in Table-II.

**Table-II:** Relation of serum PCT and GSN levels with prognosis in urosepsis patients (mg/L).

Group	1 day		3 days		5 days		7 days		n

	PCT	GSN	PCT	GSN	PCT	GSN	PCT	GSN	
the good prognosis group	4.12±1.20	187.43±15.69	2.98±1.41	157.27±14.61	2.16±0.81	112.23±15.61	1.05±0.82	52.84±13.38	54
the poor prognosis group	6.04±1.17	139.17±13.64	6.74±1.36	101.42±14.39	5.48±1.74	74.36±12.93	4.15±0.85	39.84±10.67	17
t	7.161	10.537	12.916	14.384	17.732	12.581	16.042	7.316	
p	<0.001	<0.001	<0.001	<0.001	<0.001	<0.001	<0.001	<0.001	

Patient prognosis (good prognosis vs poor prognosis) was used as the dependent variable. The serum PCT level and plasma GSN level on the 7^th^ day were used as the independent variables. After adjusting for the age and sex factors, logistic regression analysis was conducted. The results showed that PCT was an independent risk factor influencing the prognosis of urosepsis patients and that GSN was a protective factor (P<0.05), as shown in Table-III.

**Table-III:** Logistic regression analysis of factors influencing the prognosis of urosepsis patients.

Factor	β	SE	Wald χ^2^	P	OR	95%CI
PCT	0.679	0.311	8.003	0.005	2.501	1.318~3.625
GSN	-0.351	0.204	7.198	0.012	0.583	0.397~0.719

## DISCUSSION

Urosepsis is often caused by urinary tract infection or occurs after iatrogenic urinary system surgery. It has been reported that after iatrogenic surgery, the morbidity and fatality rates related to urosepsis can be as high as 23% and 35.2%, Respectively.^4^ Urosepsis is a malignant host response of the body to general infection and progresses rapidly. Once it progresses to severe septicopyemia, toxic shock or multiorgan failure, it is difficult to correct, with a very high fatality rate. At present, there is a lack of tests for early diagnosis, thus leading to a delay in diagnosis. Nonstandard use of antibiotics often results in multidrug resistance phenomena, thus increasing treatment difficulty.^5^ The gold standard for diagnosis of septicopyemia mainly depends on etiological examination. It takes 2-3 days for microculture results, which to a great extent delays early screening. Thus, seeking biological markers that can rapidly diagnose and judge urosepsis prognosis and rationally choosing antibiotic therapy are especially important for clinicians.

PCT is a calcitonin precursor protein composed of 116 amino acids. It consists of the immature calcitonin protein and the calcitonin and N-terminal residual substrate fragments. It is mainly synthesized and secreted by thyroid C cells. Under normal physiological conditions, in vivo PCT levels are very low or cannot be detected.^6^ In cases of infection and trauma, the in vivo PCT levels rise substantially and remain stable. PCT is a reliable index of severe bacterial inflammation and fungal infection, and it can reflect the degree of illness.^7^ In general infections, PCT is mainly generated by macrophages and monocytes in different organs. The research shows that as bacterial endotoxin enters the body, PCT will be synthesized within 2-3 days in the plasma. It often takes 12-48 h to increase the concentration to the maximum value. PCT has high sensitivity and specificity for general infection and is used for the early diagnosis of sepsis. In addition, it can effectively reflect the order of disease severity and has some significance in guiding disease treatment.^8^ The results of this study showed that, compared with that in the control group, the PCT level of the urosepsis group rose dramatically. PCT may participate in the pathological process of urosepsis. By comparing the poor prognosis group with the good prognosis group, it was found that the higher the serum PCT level was, the worse patient prognosis. Thus, PCT has high early diagnostic value for urosepsis, and it is a risk factor.

Normally, GSN exists in the body as a monomer or a polymer. The two forms maintain dynamic equilibrium through polymerization and depolymerization. GSN cleaves and closes actin via calcium ion dependence and adjusts actin structure and length. When there is a lack of calcium ions, GSN is in an inactive state. The segments are tightly packed together to present a sphere. When the concentration of intracellular calcium ions reaches 6~10 mol/L, calcium ions and GSN combine and GSN becomes activated, and the actin binding site is exposed. Calcium ions are bound with actin to mediate actin cleavage and closure. GSN can buffer the toxic side effects of actin released from damaged tissues and can protect the body from excessive inflammatory reactions during the pathological process of septicopyemia.^2^,^9^,^10^ The series of pathologic changes in urosepsis patients will damage the cells. A high amount of actin is released into the blood, and GSN in the blood is consumed gradually. The results of this study showed that the plasma GSN level of the urosepsis group was obviously lower than that of the control group. The plasma GSN level of the poor prognosis group was obviously lower than that of the good prognosis group. The lower the plasma GSN level was, the worse the patient prognosis was. Logistic regression analysis results indicated that PCT was an independent risk factor influencing the prognosis of urosepsis patients and that GSN was a protective factor, demonstrating that PCT and GSN can be used as predictive indices of the prognosis in urosepsis patients. However, the sample size in this study was limited, and the results also have limitations. In conclusion, serum PCT and plasma GSN levels can accurately predict the severity and prognosis of urosepsis patients and reflect the disease state of early urosepsis patients. High PCT levels and low GSN levels indicate poor prognosis, and clinicians should consider these values.

### Limitations of the study

Due to relatively small number of subjects and samples in this study, the test results may be affected to a certain extent. A prospective randomized controlled study can be further carried out in the future.

### Authors’ Contributions

**NC & ZY:** Designed this study and prepared this manuscript and are responsible and accountable for the accuracy of the work.

**ZC & NC:** Collected and analyzed clinical data.

**NC &** **ZY:** Contributed equally to this manuscript.

## References

[1] Van Nieuwkoop C, Bonten TN, van't Wout JW, Kuijper EJ, Groeneveld GH, Becker MJ (2010). Procalcitonin reflects bacteremia and bacterial load in urosepsis syndrome:a prospective observational study. Crit Care.

[2] Kustan P, Horvath-Szalai Z, Muhl D (2017). Nonconventional Markers of Sepsis. EJIFCC.

[3] Naber KG, Bergman B, Bishop MC, Bjerklund-Johansen TE, Botto H, Lobel B (2001). EAU guidelines for the management of urinary and male genital tract infections. Urinary Tract Infection (UTI) Working Group of the Health Care Office (HCO) of the European Association of Urology (EAU). Eur Urol.

[4] Gaieski DF, Edwards JM, Kallan MJ, Carr BG (2013). Benchmarking the incidence and mortality of severe sepsis in the United States. Crit Care Med.

[5] Chalupa P, Beran O, Herwald H, Kaspříková N, Holub M (2011). Evaluation of potential biomarkers for the discrimination of bacterial and viral infections. Infection.

[6] Hosokawa Y, Shimizu T, Owari T, Otsuka K, Hayashi Y, Fujimoto K (2017). Clinical Evaluation of Urosepsis in Tane General Hospital;Clinical Utility of Measurement of Procalcitonin. Hinyokika Kiyo.

[7] Zheng J, Li Q, Fu W, Ren J, Song S, Deng G (2015). Procalcitonin as an early diagnostic and monitoring tool in urosepsis following percutaneous nephrolithotomy. Urolithiasis.

[8] Canat HL, Can O, Atalay HA, Akkaş F, Otunctemur A Procalcitonin as an early indicator of urosepsis following prostate biopsy. Aging Male.

[9] Dahl B, Schiødt FV, Ott P, Wians F, Lee WM, Balko J (2003). Plasma concentration of Gc-globulin is associated with organ dysfunction and sepsis after injury. Crit Care Med.

[10] Horvath-Szalai Z, Kustán P, Szirmay B, Lakatos A, Christensen PH, Huber T (2018). Predictive value of serum gelsolin and Gc globulin in sepsis - a pilot study. Clin Chem Lab Med.

